# Methodological Approaches to Bioavailability of Oral Creatine in Randomized Controlled Trials: A Meta-Research Study

**DOI:** 10.3390/nu18162697

**Published:** 2026-08-18

**Authors:** Viljemka Bučević Popović, Antonela Sovulj, Sanda Raić, Ivana Pezelj, Malgorzata M. Bala, Livia Puljak

**Affiliations:** 1 Department of Chemistry, Faculty of Science, University of Split, 21000 Split, Croatia; 2 Department of Biology, Faculty of Science, University of Split, 21000 Split, Croatia; antonela.sovulj@pmfst.hr; 3 Mediterranean Institute for Life Sciences, University of Split, 21000 Split, Croatia; sraic@medils.unist.hr; 4 Doctoral Study of Biophysics, Faculty of Science, University of Split, 21000 Split, Croatia; izaper@pmfst.hr; 5 Chair of Epidemiology and Preventive Medicine, Department of Hygiene and Dietetics, Jagiellonian University Medical College, 31-034 Krakow, Poland; malgorzata.1.bala@gmail.com; 6 Center for Evidence-Based Medicine, Department of Nursing, School of Medicine, Catholic University of Croatia, 10000 Zagreb, Croatia; livia.puljak@gmail.com

**Keywords:** creatine, meta-research, supplement, bioavailability

## Abstract

**Background:** Creatine is one of the most widely used dietary supplements for enhancing physical performance and is increasingly investigated for a range of health-related applications. Although creatine monohydrate is the most commonly used formulation, concerns have been raised regarding its oral bioavailability, prompting the development of alternative formulations and delivery strategies. However, it is unclear to what extent randomized controlled trials (RCTs) evaluating oral creatine supplementation address issues related to bioavailability. Therefore, we aimed to assess how bioavailability of oral creatine is considered and reported in RCTs. **Methods:** We conducted a meta-research study of published RCTs in which orally administered creatine was used as an intervention or comparator, regardless of participant characteristics or outcomes. MEDLINE and Embase were searched from inception to 1 March 2024. We extracted data on trial characteristics, creatine formulations used, mentions of bioavailability, and comparisons between different creatine products. Data were summarized descriptively. **Results:** We included 357 reports corresponding to 343 RCTs published between 1994 and 2023. Creatine monohydrate was the most commonly used formulation, reported in 275 (80.2%) trials; however, 53 (15.5%) reports did not specify the exact creatine form used. Bioavailability was mentioned in only 38 (11.1%) reports, most frequently in the discussion section. Four trials (1.2%) reported specific measures intended to enhance bioavailability, including instructions regarding supplement dissolution or co-ingestion with food or carbohydrates. Five trials (1.5%) compared more than one creatine formulation, but only three (0.9%) discussed bioavailability. These formulation comparisons were frequently limited by non-equimolar dosing regimens. **Conclusions:** RCTs of oral creatine supplementation rarely address bioavailability and often provide insufficient information on the exact creatine formulation used. Few trials reported strategies to enhance bioavailability, and direct comparisons between creatine formulations were uncommon and methodologically limited, particularly because of non-equimolar dosing. Future trials should improve reporting of creatine formulations, incorporate bioavailability-related outcomes, and use appropriate dosing strategies when evaluating alternative creatine products.

## 1. Introduction

Creatine is a naturally occurring amino acid derivative with an important role in energy metabolism. In the human body, the energy generated through nutrient oxidation is mainly stored and utilized as ATP, but the amount of this high-energy molecule is depleted within seconds of high-intensity activity [1]. Creatine, which is mostly present in the form of phosphocreatine, serves to rapidly regenerate ATP from ADP and phosphate (Pi). Consequently, the main creatine stores are found in tissues that have a high and fluctuating need for ATP such as skeletal or cardiac muscle and brain [2,3].

The daily need for creatine for an average person is ~2–4 g [4]. Approximately half of this amount is acquired by endogenous synthesis using amino acids arginine and glycine as starting material. The first biosynthetic step yields guanidinoacetate which is then methylated using S-adenosylmethionine to form creatine. Creatine is subsequently phosphorylated by creatine kinase into phosphocreatine, the physiologically relevant form [3]. The remainder of required daily needs is acquired through the diet (exogenous sources), predominantly from dietary meat and fish [2,4].

In the past decades, creatine nutritional supplements emerged as alternative sources of creatine to boost body stores and increase muscle performance, particularly in sports that require short, explosive bursts of power and strength [3]. During that period, creatine became one of the most studied and most widely used supplements in the field of sport nutrition [5,6]. In recent years, studies have reported additional health benefits of creatine beyond its usage in sports. The effects of creatine were explored in various human diseases and age-related health conditions [6,7]. For example, multiple studies have explored the benefits of creatine to improve cognition [8], women’s health [9], glucose management and diabetes [10], vascular health [11] and immune responses [12].

While supplementation can effectively increase nutrient body stores, not all of the ingested compound ultimately reaches the target cells. In pharmacology, oral drug bioavailability is defined as the fraction of an active compound that reaches systemic circulation. It is typically assessed through pharmacokinetic studies evaluating four main parameters: absorption, distribution, metabolism, and excretion (ADME) [13]. Likewise, in nutrition science, bioavailability describes the proportion of a nutrient absorbed and transported in a form that the body can use or store [14]. Factors such as poor solubility, chemical instability, degradative metabolism within the gastrointestinal tract, and limited transport across the intestinal epithelial membranes frequently reduce the bioavailability of dietary supplements. Since, except in specific cases [15], there are no regulatory requirements to provide data on supplement bioavailability before a product is placed on the market, this is often an understudied area.

The most commonly used creatine supplement is, by far, creatine monohydrate [2]. It remains the most extensively investigated creatine formulation and continues to represent the reference standard in both experimental and applied sports nutrition research. Recent evidence also reinforces its role as an effective supplement for muscle hypertrophy primarily through phosphocreatine buffering, thereby supporting volume and quality of training [16]. However, research indicates that creatine monohydrate, while considered ‘the golden standard’ for creatine supplementation may have limitations in bioavailability, primarily related to its limited solubility [17]. Thus, novel formulations were developed with the intent to obtain products of improved efficacy [1,2,18].

In line with this goal, Alraddadi et al. demonstrated that creatine hydrochloride, which possesses significantly higher aqueous solubility than creatine monohydrate, exhibited a notable increase in oral bioavailability [19]. Moreover, the stability of creatine derivatives during oral administration and digestion is also a vital consideration. Generally, creatine is converted to creatinine by intramolecular cyclization. Hageböck et al. highlighted that certain derivatives, such as creatine ethyl ester, are unstable under physiological conditions as they hydrolyze rapidly and degrade to creatinine, which could adversely affect bioavailability [20]. It has been documented that transformation of creatine to creatinine is increased at low pH, which was a rationale behind marketing pH-stabilized creatine sources such as buffered creatine monohydrate [1]. The effect of the physiochemical properties of creatine and its various forms on solubility, permeability and overall pharmacokinetic profile was emphasized in the recent review by Kreider et al., which also noted that many marketed alternatives to creatine monohydrate have not demonstrated superior bioavailability or effectiveness [2].

We have shown previously that the issue of bioavailability of bioactive compounds is not necessarily well addressed in the highest level of evidence in medicine. Our studies of curcumin, which is characterized by low oral bioavailability, have shown that researchers rarely address and incorporate in the methodology the issue of bioavailability when conducting clinical trials and systematic reviews [21,22]. We were unable to find such methodological studies for creatine supplements in the available literature, therefore we planned this study to address this research gap.

The aim of this meta-research study was to analyze whether randomized controlled trials (RCTs) on creatine address bioavailability of creatine supplements, whether trialists implemented strategies that could improve bioavailability, and whether they discussed their findings in context of creatine bioavailability. To obtain a comprehensive insight of how bioavailability is assessed in RCTs, our screening strategy included a broader set of terms related to its contributing factors and underlying mechanisms.

## 2. Materials and Methods

### 2.1. Study Design

This study is a meta-research which analyzed published RCTs. Since this study analyzed publicly available research articles, approval of a research protocol by an institutional ethics committee was not applicable.

### 2.2. Protocol

The study protocol was designed prospectively. The protocol is available in Supplementary Information S1.

### 2.3. Inclusion Criteria

We included RCTs published as full reports in the English language with results in scholarly journals where creatine was administered orally for systemic absorption, analyzed as an intervention or a comparator, irrespective of the type of participants and outcomes that were used in a trial. Protocols of RCTs, if they were published as a full-text article in a scholarly journal, were also included. We also included manuscripts that reported post hoc analyses of already published RCTs. When we found multiple reports for a single study, we collated them with the main publication.

### 2.4. Exclusion Criteria

Studies using mixtures of creatine with other compounds were excluded unless other bioactive compounds were administered to both the intervention and control groups, or additional compounds were added specifically for bioavailability enhancement.

Studies utilizing non-oral routes of creatine administration for systemic absorption were also excluded. We excluded in vitro, in vivo and in silico studies. We excluded studies published as conference abstracts, results published in editorials and short reports (i.e., not full-text articles in scholarly journals). We excluded studies available only as records on trial registry without full journal publication of the protocol.

### 2.5. Search and Screening

A search of MEDLINE and Embase via Ovid was conducted on 1 March 2024 by using the following search syntax: creatine OR creatinol OR creatyl. All bibliographic records found with this search were retrieved. We used Covidence online platform (www.covidence.org) for screening of titles/abstracts and full texts. We uploaded search results from all sources and removed duplicates using the Covidence algorithm. Subsequently, the studies that were not RCTs were filtered out by the Cochrane Randomized Controlled Trial (RCT) classifier built into Covidence. The classifier assesses all the studies imported into Covidence and automatically assigns one of two tags: “Possible RCT” or “Not RCT”. We excluded records classified as “not RCT” and then we screened all the records classified with the “Possible RCT” tag to assess whether they met the inclusion criteria.

In the first stage of screening, two authors individually screened titles and abstracts of all records in Covidence to verify that they indeed fulfill inclusion criteria. In the second stage, full texts of all records that were considered eligible or potentially eligible during the first screening stage, were assessed for their eligibility against inclusion criteria. We used full texts that were added by Covidence automated process, if available, or retrieved manually. Each full text was independently screened by two authors in Covidence. Reasons for records exclusion were noted. Two authors resolved any full-text screening disagreements through discussion, involving a third author if they could not reach a consensus.

### 2.6. Data Extraction

Data extraction was done using Covidence systematic review tool. Two authors participated in data extraction for each study and independently extracted all data. Any disagreements in extracted data were resolved via consensus among the author team.

For each included RCT, we extracted the following data: first author’s last name, publication year, study title, trial registration number, number of randomized participants, number of study arms, duration of follow-up, participants characteristics, and type of creatine product evaluated (either as an intervention or a comparator) along with the complete description of creatine from the Section 2. Any mentions of creatine bioavailability throughout the manuscript were also extracted, noting the specific manuscript section where considerations regarding creatine bioavailability occurred. To ensure that all relevant information regarding the bioavailability of creatine supplements were extracted, publications were also screened for mentions of related terms such as bioenhancement, absorption, transportability or uptake. Because the objective of this study was to explore how trialists addressed and reported bioavailability, we did not apply a predefined definition of bioavailability. Instead, we extracted verbatim all statements referring to bioavailability or related concepts (e.g., absorption, uptake, transport, pharmacokinetics, or bioenhancement) and categorized them inductively according to their content.

For the formulation of creatine that was used, both commercial and generic names of the product were extracted, if reported in the article.

For matching pairs of published RCTs and their corresponding protocols, we compared whether there were any differences in the description of creatine, or descriptions of its bioavailability.

After data extraction, we categorized responses and presented the data narratively. Characteristics of publications were summarized at the report level, whereas intervention-related characteristics and bioavailability reporting were summarized at the study level, with information collated across multiple reports of the same study when applicable.

### 2.7. Statistics

Data were given as numbers and frequencies, median and interquartile range. We used Microsoft Excel (Microsoft Inc., Redmond, WA, USA) for the analyses.

## 3. Results

**Figure 1:**
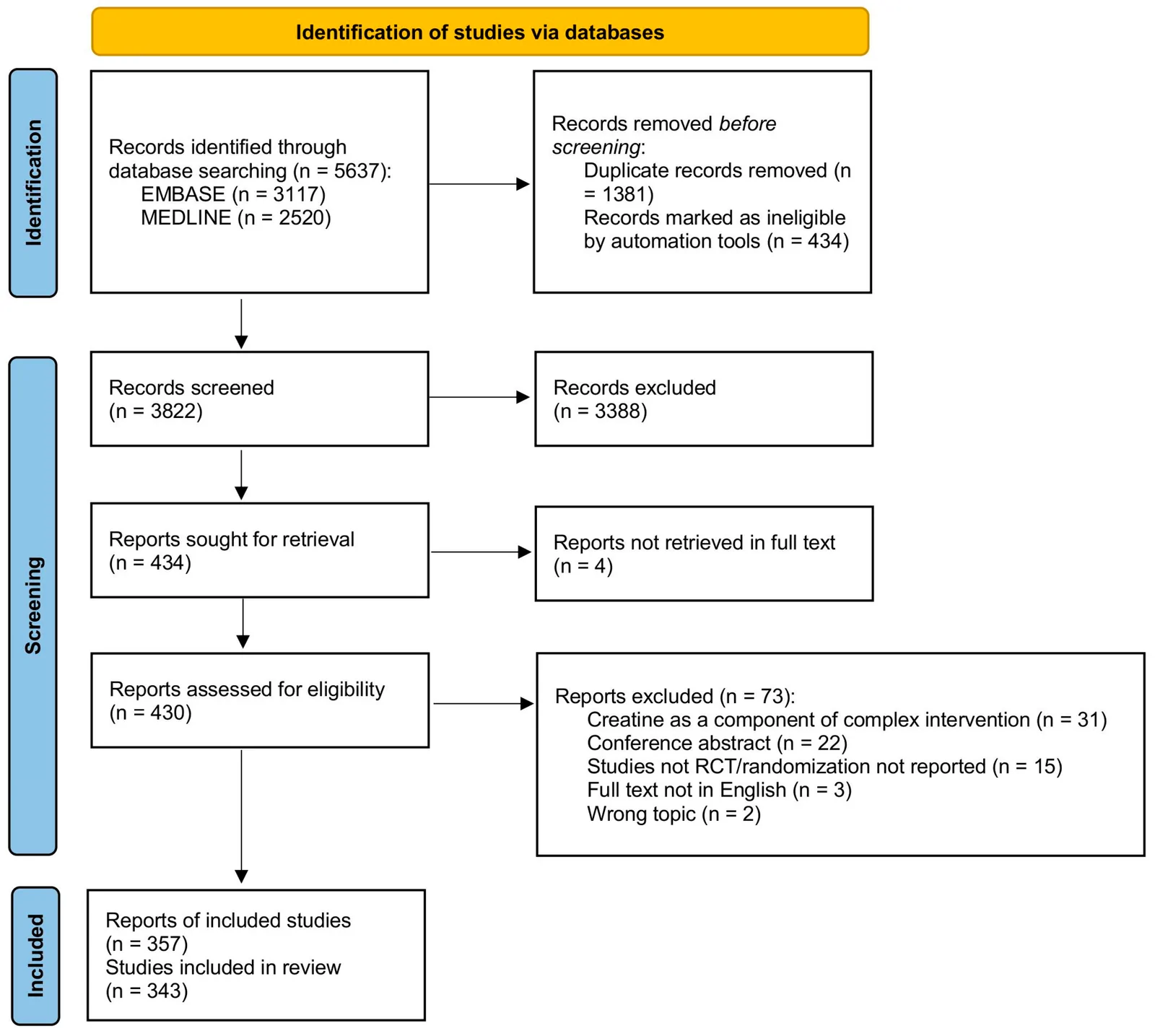
Flowchart of study selection according to PRISMA (Preferred Reporting Items for Systematic Reviews and Meta-Analysis) guidelines.

We retrieved 5637 records via search. After screening of titles and abstracts, 434 records were assigned for full text examination. We excluded 73 records that were not eligible due to reasons listed in Supplementary Table S1. Main reasons for exclusion were records on the usage of creatine in complex interventions (combination of creatine and other compounds was examined), records that were conference abstracts or trials not meeting RCT criteria. We included 357 records in the analysis, that yielded 343 distinct studies after collating multiple papers from the same trial. The list of included studies is reported in Supplementary Table S2. A flow diagram of the study selection is shown in Figure 1.

### 3.1. Characteristics of Included Trials

The included RCTs were published between the years 1994 and 2023. Characteristics of included trials analyzed using individual publication as the unit of analysis are shown in Table 1. Trials were published by 127 journals, most frequently in the journals *Medicine and Science in Sports and Exercise*, and *Journal of Strength and Conditioning Research* (Table 1). Protocol registration was reported in 65 (18.2%) articles. The median number of participants randomized was 24. The median number of study arms was two. The median duration of patient follow-up was 4 weeks (Table 1).

The most common categories of included participants were healthy participants, followed by athletes, trained/recreationally active individuals and older/elderly individuals. Collectively, these four categories represent approximately two-thirds of all reports (Table 1). The remaining categories comprise various populations, many of which include patients with diseases or injuries of the nervous system. All participant categories are shown in Supplementary Table S3.

The 357 included publications were mapped to 343 independent studies and creatine interventions were analyzed using study as the unit of analysis. The most common category was creatine monohydrate which was used in 80.2% of 343 included trials (Table 2). However, the reports differed widely in the level of details they reported on the creatine monohydrate product. In many instances, any information regarding purity or manufacturer of creatine supplement was lacking. As much as 15.5% of studies simply used the term ‘creatine’ without specifying additional information that would enable unambiguous categorization of a supplement as anhydrous creatine. Regarding the widespread use of creatine monohydrate, it is likely that most of these trials are in fact using creatine monohydrate as their creatine form. Other types of creatine formulations in analyzed publications are listed in Table 2. The list does not include formulations where creatine was mixed with other compounds and therefore represent a complex intervention not meeting our inclusion criteria. We found only five reports that compared two creatine forms, namely, creatine monohydrate with creatine citrate [23], magnesium creatine chelate [24], creatyl-l-leucine [25] creatine nitrate [26], or polyethylene glycosylated creatine hydrochloride [27]. The complete description of creatine supplements (commercial name, manufacturer, etc.), where available, as well as doses used in the trials are reported in Supplementary Table S3.

### 3.2. Mentions of Bioavailability

Among the 343 included RCTs, only 38 (11.1%) had mentioned the bioavailability of creatine throughout the manuscript, irrespective of the context. Manuscript parts where this was most commonly mentioned was Discussion, where 28 (8.2%) of the included trials mentioned bioavailability (Table 3). The extracted statements covered a broad range of concepts, including gastrointestinal absorption, circulating creatine concentrations, tissue uptake, pharmacokinetics, urinary excretion, and claims regarding the performance of specific formulations. Supplementary Table S3 presents verbatim extractions of those text sections where creatine bioavailability was mentioned.

Among the 38 trials that mentioned bioavailability in the manuscript, 10 trials reported the use of alternative creatine supplements in addition to or instead of the standard creatine form, i.e., creatine monohydrate. Creatine citrate was the only formulation among those listed in Table 2 that was not used in any of the trials mentioning bioavailability. Four trials reported methods to enhance bioavailability in the form of specific instructions on the mode of usage to facilitate intestinal absorption and muscle uptake. Participants were advised to dissolve the supplement in an adequate amount of (warm) water or ingest the creatine supplement together with food or maltodextrin [28,29,30,31].

Among trials included in this study, five (1.5%) compared different creatine formulations but only three (0.9%) discussed bioavailability and were therefore analyzed in greater detail (Supplementary Table S4). Each of them compared the most commonly used form, creatine monohydrate, with alternative creatine supplements, namely, creatyl-l-leucine [25], creatine nitrate [26] or polyethylene glycosylated (PEG) creatine hydrochloride [27]. In all these trials, significant increase in creatine muscle stores or power were observed only in the creatine monohydrate group. The study by Galvan et al. [26] was the only one that reported data on creatine serum concentration which was increased after 7-day loading with creatine monohydrate or two creatine nitrate doses (low and high) compared to placebo. At the end of the 28-day supplementation period, plasma creatine remained significantly elevated only in the high creatine nitrate group. However, the dosing regimen used in these studies did not provide equimolar quantities of creatine in compared supplementation groups, as they were focused on comparing different supplementation regimens rather than comparative bioavailability assessment. For example, one of the studies conducted by Galvan et al. [26] compared maintenance supplementation with 3 g/day of creatine monohydrate to 1.5 g/day (low dose) or 3 g/day (high dose) of creatine nitrate. These dosing regimens translate to the daily doses of 20.11 mmol of creatine supplied in the form of creatine monohydrate vs. 7.73 (low dose) or 15.45 mmol (high dose) of creatine provided as creatine nitrate. Consequently, the participants in the creatine monohydrate group received 2.6- and 1.3-fold higher amounts of creatine in the low and high creatine nitrate group, respectively. Taken together, the participants in the creatine monohydrate group received higher molar quantities of creatine in all the trials: 1.6× more compared to creatyl-l-leucine [25], 1.3–4.3× more than creatine nitrate [26] and 2× or 4× more than PEG-creatine hydrochloride [27] (Supplementary Table S5).

The vast majority of trials addressing creatine bioavailability aimed to increase muscle creatine content, whereas in only two studies the goal was to enhance creatine concentrations within the brain [32,33]. Most of the trials (97%) that addressed oral bioavailability of creatine were published from 2000 onwards.

## 4. Discussion

Bioavailability is a critical factor in determining the effectiveness of dietary supplements, including creatine, as it influences the extent and rate at which the active ingredient is absorbed and becomes available at the site of action [2]. Our study showed that among 343 RCTs that analyzed the effect of oral creatine supplementation, only 38 (11.1%) addressed the issue of creatine bioavailability. Among those, only three trials (0.9%) compared the efficacy of two types of creatine supplements head-to-tail.

As anticipated, a vast majority of RCTs included in our analysis (80.2%) used creatine monohydrate as a creatine supplement (Table 2). A concerningly large number of studies (15.5%) failed to specify the exact chemical form of creatine supplement. Due to the prevalent use of creatine monohydrate, it may be assumed that the authors are in fact referring to creatine monohydrate, bringing the total proportion of trials using this form to over 95%. However, to improve methodological transparency, future studies should specify the exact chemical composition of the tested supplement, as this information is crucial for comparison of outcomes across different protocols.

In terms of methodological approaches to improve bioavailability, some studies have explored the use of supplements with improved solubility or specific delivery systems. Examples of supplements with enhanced solubility are creatine salts such as creatine citrate [23,34,35,36,37,38,39,40] and creatine pyruvate [41]. It should be noted that these supplements deliver creatine together with another nutrient which by itself may have ergogenic properties or act synergistically with creatine [2]. Therefore, the overall beneficial effects may stem from the characteristics of the anion, rather than improvements in creatine bioavailability when delivered in salt form. To accurately assess whether a specific formulation offers superior bioavailability, trialists should utilize controls that account for the non-creatine components of the supplement.

In the domain of delivery systems, studies have investigated the effects of PEGylation, where creatine is attached to a water-soluble polymer polyethylene glycol [27,42,43]. This strategy is commonly used and clinically proven to enhance the solubility and half-life time of the elimination of pharmaceuticals [44]. As reported in the study by Herda et al. [27], administration of PEGylated creatine leads to comparable increases in muscle strength as creatine monohydrate, but with reduced doses. The study by Fielding et al. investigated the effects of lipid multi-particulate formulations and concluded that this approach enhances serum bioavailability of creatine [45]. Such innovative methodologies could be pivotal in enhancing the absorption and efficacy of creatine supplements, yet they are often underrepresented in RCTs.

Among studies that met our inclusion criteria and addressed bioavailability, only three of them compared multiple creatine products. Namely, the efficacy of creatine monohydrate in improving muscle creatine content or functional parameters was compared to alternative formulations: creatyl-l-leucine [25], creatine nitrate [26] and polyethylene glycosylated creatine hydrochloride [27]. However, these studies were primarily focused on assessment of different supplementation regimens rather than supplement bioavailability and did not utilize equimolar creatine dosing in their supplementation protocol. Therefore, their design precludes valid inference regarding comparative creatine bioavailability (Supplementary Tables S4 and S5). Any reported advantages of superior formulation may be simply an artifact of higher absolute creatine intake rather than enhanced pharmacokinetic profile. To address these limitations, future research aimed at comparing bioavailability must employ equimolar dosing protocols to ensure that any observed differences in efficacy are attributable to improvements in bioavailability and target tissue uptake.

In our analysis, we have found only 38 studies (11.1%) that mention bioavailability in any way, suggesting that most of the included creatine supplementation RCTs were designed primarily to evaluate performance and clinical outcomes of the intervention. Among those that mentioned bioavailability, circulatory creatine concentration, which provides pharmacokinetic information relevant to systemic exposure, was reported in the results and discussed in a limited number of studies (six and seven studies, respectively). It may be assumed that the majority of studies do not address the bioavailability of creatine because this does not represent the most critical factor for effective supplementation. The entry of creatine into target tissues (primarily skeletal muscle, but also the brain and heart) is a highly regulated process that relies on a specific transport system. The primary gateway for creatine is sodium- and chloride-dependent creatine transporter 1 (CT1 or SLC6A8) which is responsive to insulin and the amount of creatine already inside the cell [46,47]. Once the cellular creatine stores are full, any excess creatine in the bloodstream will not be taken up by the tissue and instead creatine or its degradation products will be excreted via the kidney. Therefore, the researchers have focused less on bioavailability and more on the restricted uptake into the target site, mainly, muscle cells. A commonly used strategies to enhance uptake include co-ingestion with carbohydrates or other insulin-sensitizing nutrients or the timing of ingestion relative to exercise, although the evidence for the latter remains equivocal [5].

Among studies that did address creatine bioavailability, only two trials focused on enrichment of brain creatine content [32,33], indicating that this is an understudied research area. Previous studies showed that creatine monohydrate supplementation increases brain creatine levels by 5–15%, compared with 20–40% in the muscle [6,48]. This relatively low response to creatine supplementation in the brain might be explained by the lack of sufficient number of SLC6A8 transporters at the blood–brain barrier [48]. More permeable forms of creatine, which cross membranes via passive diffusion, would facilitate both intestinal absorption and uptake into the brain and allow for more effective treatment of neurological conditions without requiring extremely high doses over extended periods. Therefore, brain creatine uptake represents the research area where the exploration of bioavailability-enhancing strategies could be more thoroughly integrated and possibly holds the greatest clinical promise.

Lastly, the overall findings of this study corroborate the conclusions made by other investigators who pinpointed that the marketing claims about purportedly enhanced bioavailability of new creatine formulations are not adequately supported by evidence from clinical trials [2,49].

### 4.1. Limitations

This study had several potential limitations. First, design-related limitations of the review itself should be acknowledged. The review was conducted according to a prospectively defined protocol using MEDLINE and Embase as the primary literature sources. Although these databases capture the majority of biomedical and sports medicine research, relevant studies indexed exclusively in other databases (e.g., Web of Science or Scopus) may not have been identified. Furthermore, because the objective of the present work was to provide a methodological appraisal rather than a quantitative estimate of treatment effects, no meta-analysis or formal quantitative synthesis was performed. Consequently, the findings should be interpreted as a qualitative evaluation of methodological practices rather than as pooled estimates of comparative efficacy or bioavailability.

Second, several operational choices were made to address the specific objective of the review. Only randomized controlled trials comparing orally administered creatine formulations were considered eligible, and the review focused specifically on methodological aspects relevant to comparative oral bioavailability. Studies investigating creatine efficacy without formulation comparisons, non-randomized designs, or mechanistic investigations without comparative clinical data were therefore intentionally excluded. These decisions were made to maintain methodological consistency and should not be interpreted as judgments regarding the overall quality or clinical relevance of those studies.

Finally, the available body of evidence itself has important limitations. Considerable heterogeneity existed across the included studies with respect to participant characteristics, creatine formulations, dosing regimens, intervention duration, comparator selection, and outcome assessment. In addition, many studies lacked methodological features necessary for valid comparisons of oral bioavailability, including equimolar dosing strategies and pharmacokinetic outcome measures. This heterogeneity limited direct comparisons between studies and prevented conclusions regarding the relative oral bioavailability of many commercially available creatine formulations. Collectively, these findings emphasize the need for future RCTs employing standardized methodology, appropriate pharmacokinetic endpoints, and transparent reporting to permit robust comparisons of creatine bioavailability.

### 4.2. Future Research

Future studies should improve methodological transparency by always specifying the exact chemical form and implementing equimolar dosing regimens alongside appropriate control groups to account for non-creatine components of the supplement. In addition, pharmacokinetic measures, such as circulatory creatine concentrations, should be tracked as many RCTs focus on performance outcomes without adequately linking these results back to direct bioavailability metrics.

Although creatine supplementation has traditionally been used in the field of sport nutrition to increase performance in recreational or professional athletes, the research interest diverged into application of creatine as a therapeutic agent in elderly and specific patient populations. While creatine is well tolerated in healthy individuals [49], individuals with pre-existing health conditions might be more at risk to experience side-effects after long-term creatine use. The included trials were generally short, with a median follow-up of 4 weeks (IQR 9 weeks). It is necessary to conduct long-term RCTs to assess the sustained benefits and potential adverse effects of chronic creatine use. Novel delivery methods should be explored particularly for central nervous system disorders where bioavailability-optimized formulations hold the greatest therapeutic potential.

## 5. Conclusions

Randomized controlled trials of oral creatine supplementation rarely address bioavailability and often provide insufficient information on the exact creatine formulation used. Few trials reported strategies to enhance bioavailability, and direct comparisons between creatine formulations were uncommon and methodologically limited, particularly because of non-equimolar dosing. Future trials should improve reporting of creatine formulations, incorporate bioavailability-related outcomes, and use appropriate dosing strategies when evaluating alternative creatine products.

## Figures and Tables

**Table 1:** Characteristics of included randomized controlled trials. Data were analyzed at the record level (N = 357).

Characteristics	Values
The most common journals where trials were published, N (%)	
*Medicine and Science in Sports and Exercise*	37 (10.3)
*Journal of Strength and Conditioning Research*	36 (10.1)
*International Journal of Sport Nutrition and Exercise Metabolism*	20 (5.6)
*The Journal of Sports Medicine and Physical Fitness*	17 (4.8)
*Nutrients*	14 (3.9)
*European Journal of Applied Physiology*	12 (3.3)
*Journal of Applied Physiology (Bethesda, Md.: 1985)*	12 (3.3)
*Neurology*	11 (3.1)
Other	198 (55.5)
Trial registered, N (%)	
Yes	65 (18.2)
Not reported	292 (81.8)
Number of participants randomized, median (IQR)	24 (22)
Number of study arms, median (IQR)	2 (0)
Duration of participant follow-up in weeks, median (IQR)	4 (9)
The most common categories of included participants, N (%)	
Healthy participants	76 (21.2)
Athletes	74 (20.7)
Trained/recreationally active individuals	63 (17.6)
Older/elderly individuals	32 (9.0)
Untrained/inactive individuals	9 (2.5)
Patients with Parkinson disease	9 (2.5)
Patients with muscular dystrophies	8 (2.2)
Arsenic-exposed individuals	6 (1.7)
Patients with major depressive/bipolar disorder	6 (1.7)
Amyotrophic lateral sclerosis (ALS) patients	5 (1.4)
Type 2 diabetes patients	5 (1.4)
Cancer patients	4 (1.1)
Army-duty subjects	3 (0.8)
Cardiac/coronary artery disease patients	3 (0.8)
Hemodialysis patients	3 (0.8)
Huntington disease patients	3 (0.8)
Individuals with osteoarthritis	3 (0.8)
Moderately active individuals	3 (0.8)
Patients with chronic obstructive pulmonary disease (COPD)	3 (0.8)
Patients with mitochondrial diseases	3 (0.8)
Spinal cord injury patients	3 (0.8)
Other	33 (9.2)

IQR = interquartile range.

**Table 2:** The forms of creatine most commonly used in analyzed trials. Multiple reports from the same study were collated and data were analyzed at the study level (N = 343) *.

Creatine Form	N (%)
Creatine monohydrate	275 (80.2)
Creatine, *unspecified*	53 (15.5)
Creatine citrate	8 (2.3)
Polyethylene glycosylated (PEG) forms of creatine or creatine monohydrate/hydrochloride	3 (0.9)
Creatine nitrate	2 (0.6)
Magnesium creatine chelate	2 (0.6)
Anhydrous creatine	1 (0.3)
Creatyl-l-leucine	1 (0.3)
Creatine pyruvate	1 (0.3)
Creatine monohydrate in a gel matrix Actijube	1 (0.3)
Lipid multi-particulate formulated creatine monohydrate	1 (0.3)

* The numbers of included creatine forms add up to 348 since five trials included two creatine forms.

**Table 3:** Mentions of bioavailability found in 38 included trials. Multiple reports from the same study were collated and data were analyzed at the study level (N = 343).

Study Section	Number of Studies Mentioning Bioavailability (%)	Categorization	N
Abstract	3 (0.9)	Lack of effects of creatine despite evidence of absorption	2
		Assessment of bioavailability stated as study topic	1
Introduction *	13 (3.8)	Bioavailability may vary depending on the type of supplement	9
		General note on creatine bioavailability	2
		Efficacy of intestinal absorption of creatine	2
		Creatine plasma concentrations are reported	2
		Pharmacokinetics of creatine formulation	1
Methods	7 (2.0)	Instruction for mode of supplement intake to facilitate intestinal absorption	4
		Description of methodology used to assess bioavailability	1
		Description of methods used to measure concentration	1
		Comment on correlation of ingested creatine with its concentrations in plasma	1
Results	6 (1.7)	Creatine serum concentration following supplementation are reported	6
Discussion **	28 (8.2)	Results on serum/plasma creatine concentrations were discussed	7
		Bioavailability may vary depending on the creatine formulation	6
		The addition of carbohydrates facilitates gastrointestinal absorption of creatine	4
		Bioavailability of creatine is discussed based on urinary excretion of its metabolites	3
		General comment on creatine bioavailability	3
		Creatine absorption kinetics is discussed	3
		Intestinal absorption may be related to supplement solubility	2
		Bioavailability is deduced from the amount of creatine retained in the body	1
		The efficacy of intestinal absorption of creatine is discussed	1
		Creatine bioavailability may be species specific	1
		Pharmacokinetics of creatine is discussed	1
		Benefits of alternative forms of creatine are questioned	1
Conclusions	6 (1.7)	Recapitulation of creatine pharmacokinetics results	2
		Results indicate increased bioavailability/muscle uptake of novel creatine formulation	2
		Further research is needed to evaluate novel creatine formulation	2

* In 13 studies mentioning bioavailability, 16 distinct categories were identified for this data item. ** In 28 studies mentioning bioavailability, 33 distinct categories were identified for this data item.

## Data Availability

All raw data collected within the study are available in Supplementary Table S3.

## Supplementary Materials

The following supporting information can be downloaded at https://www.mdpi.com/article/10.3390/nu18162697/s1. Supplementary Information S1: Study protocol (the protocol contains literature references including also reference to Cooper et al. [50]); Supplementary Table S1: List of excluded studies; Supplementary Table S2: List of included studies; Supplementary Table S3: Extracted data from the included studies; Supplementary Table S4: Studies using multiple creatine formulations; Supplementary Table S5: Molar quantities in studies using compared formulations.

## References

[1] Vranes M.B., Popovic S., Ostojic S.M. (2015). New Forms of Creatine in Human Nutrition. Human Health and Nutrition: New Research.

[2] Kreider R.B., Jäger R., Purpura M. (2022). Bioavailability, Efficacy, Safety, and Regulatory Status of Creatine and Related Compounds: A Critical Review. Nutrients.

[3] Bonilla D.A., Kreider R.B., Stout J.R., Forero D.A., Kerksick C.M., Roberts M.D., Rawson E.S. (2021). Metabolic Basis of Creatine in Health and Disease: A Bioinformatics-Assisted Review. Nutrients.

[4] Kreider R.B., Jagim A.R., Antonio J., Kalman D.S., Kerksick C.M., Stout J.R., Wildman R., Collins R., Bonilla D.A. (2025). Creatine Supplementation Is Safe, Beneficial throughout the Lifespan, and Should Not Be Restricted. Front. Nutr..

[5] Antonio J., Brown A.F., Candow D.G., Chilibeck P.D., Ellery S.J., Forbes S.C., Gualano B., Jagim A.R., Kerksick C., Kreider R.B. (2025). Part II. Common Questions and Misconceptions about Creatine Supplementation: What Does the Scientific Evidence Really Show?. J. Int. Soc. Sports Nutr..

[6] Kreider R.B., Stout J.R. (2021). Creatine in Health and Disease. Nutrients.

[7] Gutiérrez-Hellín J., Del Coso J., Franco-Andrés A., Gamonales J.M., Espada M.C., González-García J., López-Moreno M., Varillas-Delgado D. (2024). Creatine Supplementation Beyond Athletics: Benefits of Different Types of Creatine for Women, Vegans, and Clinical Populations—A Narrative Review. Nutrients.

[8] Smith A.N., Morris J.K., Carbuhn A.F., Herda T.J., Keller J.E., Sullivan D.K., Taylor M.K. (2023). Creatine as a Therapeutic Target in Alzheimer’s Disease. Curr. Dev. Nutr..

[9] Smith-Ryan A.E., Cabre H.E., Eckerson J.M., Candow D.G. (2021). Creatine Supplementation in Women’s Health: A Lifespan Perspective. Nutrients.

[10] Solis M.Y., Artioli G.G., Gualano B. (2021). Potential of Creatine in Glucose Management and Diabetes. Nutrients.

[11] Clarke H., Hickner R.C., Ormsbee M.J. (2021). The Potential Role of Creatine in Vascular Health. Nutrients.

[12] Bredahl E.C., Eckerson J.M., Tracy S.M., McDonald T.L., Drescher K.M. (2021). The Role of Creatine in the Development and Activation of Immune Responses. Nutrients.

[13] Stielow M., Witczyńska A., Kubryń N., Fijałkowski Ł., Nowaczyk J., Nowaczyk A. (2023). The Bioavailability of Drugs-The Current State of Knowledge. Molecules.

[14] Richards J.D., Cori H., Rahn M., Finn K., Bárcena J., Kanellopoulos A.K., Péter S., Spooren A. (2025). Micronutrient bioavailability: Concepts, influencing factors, and strategies for improvement. Front. Nutr..

[15] Turck D., Bohn T., Castenmiller J., de Henauw S., Hirsch-Ernst K.I., Knutsen H.K., Maciuk A., Mangelsdorf I., McArdle H.J., EFSA Panel on Nutrition, Novel Foods and Food Allergens (2024). Guidance on scientific principles and data requirements for the safety and relative bioavailability assessment of new micronutrient sources. EFSA J..

[16] Mănescu A.M., Hangu S.Ș., Mănescu D.C. (2025). Nutritional Supplements for Muscle Hypertrophy: Mechanisms and Morphology-Focused Evidence. Nutrients.

[17] Miller D.W., Augustine S., Robinson D.H., Vennerstrom J.L., Wagner J.C., Bagchi D., Nair S., Sen C.K. (2013). Oral Bioavailability of Creatine Supplements. Nutrition and Enhanced Sports Performance.

[18] Fazio C., Elder C.L., Harris M.M. (2022). Efficacy of Alternative Forms of Creatine Supplementation on Improving Performance and Body Composition in Healthy Subjects: A Systematic Review. J. Strength Cond. Res..

[19] Alraddadi E.A., Lillico R., Vennerstrom J.L., Lakowski T.M., Miller D.W. (2018). Absolute Oral Bioavailability of Creatine Monohydrate in Rats: Debunking a Myth. Pharmaceutics.

[20] Hageböck M., Stahl U., Bader J. (2014). Stability of Creatine Derivatives during Simulated Digestion in an in Vitro Model. Food Funct..

[21] Mimica B., Bučević Popović V., Banjari I., Jeličić Kadić A., Puljak L. (2022). Methods Used for Enhancing the Bioavailability of Oral Curcumin in Randomized Controlled Trials: A Meta-Research Study. Pharmaceuticals.

[22] Bučević Popović V., Karahmet Farhat E., Banjari I., Jeličić Kadić A., Puljak L. (2024). Bioavailability of Oral Curcumin in Systematic Reviews: A Methodological Study. Pharmaceuticals.

[23] Derave W., Eijnde B.O., Verbessem P., Ramaekers M., Van Leemputte M., Richter E.A., Hespel P. (2003). Combined Creatine and Protein Supplementation in Conjunction with Resistance Training Promotes Muscle GLUT-4 Content and Glucose Tolerance in Humans. J. Appl. Physiol..

[24] Selsby J.T., DiSilvestro R.A., Devor S.T. (2004). Mg^2+^-Creatine Chelate and a Low-Dose Creatine Supplementation Regimen Improve Exercise Performance. J. Strength Cond. Res..

[25] Askow A.T., Paulussen K.J.M., McKenna C.F., Salvador A.F., Scaroni S.E., Hamann J.S., Ulanov A.V., Li Z., Paluska S.A., Beaudry K.M. (2022). Creatine Monohydrate Supplementation, but Not Creatyl-L-Leucine, Increased Muscle Creatine Content in Healthy Young Adults: A Double-Blind Randomized Controlled Trial. Int. J. Sport Nutr. Exerc. Metab..

[26] Galvan E., Walker D.K., Simbo S.Y., Dalton R., Levers K., O’Connor A., Goodenough C., Barringer N.D., Greenwood M., Rasmussen C. (2016). Acute and Chronic Safety and Efficacy of Dose Dependent Creatine Nitrate Supplementation and Exercise Performance. J. Int. Soc. Sports Nutr..

[27] Herda T.J., Beck T.W., Ryan E.D., Smith A.E., Walter A.A., Hartman M.J., Stout J.R., Cramer J.T. (2009). Effects of Creatine Monohydrate and Polyethylene Glycosylated Creatine Supplementation on Muscular Strength, Endurance, and Power Output. J. Strength Cond. Res..

[28] Ahmun R.P., Tong R.J., Grimshaw P.N. (2005). The Effects of Acute Creatine Supplementation on Multiple Sprint Cycling and Running Performance in Rugby Players. J. Strength Cond. Res..

[29] Connell N.J., Doligkeit D., Andriessen C., Kornips-Moonen E., Bruls Y.M.H., Schrauwen-Hinderling V.B., van de Weijer T., van Marken-Lichtenbelt W.D., Havekes B., Kazak L. (2021). No Evidence for Brown Adipose Tissue Activation after Creatine Supplementation in Adult Vegetarians. Nat. Metab..

[30] Fransen J.C., Zuhl M., Kerksick C.M., Cole N., Altobelli S., Kuethe D.O., Schneider S. (2015). Impact of Creatine on Muscle Performance and Phosphagen Stores after Immobilization. Eur. J. Appl. Physiol..

[31] Marini A.C.B., Motobu R.D., Freitas A.T.V., Mota J.F., Wall B.T., Pichard C., Laviano A., Pimentel G.D. (2020). Short-Term Creatine Supplementation May Alleviate the Malnutrition-Inflammation Score and Lean Body Mass Loss in Hemodialysis Patients: A Pilot Randomized Placebo-Controlled Trial. JPEN J. Parenter. Enter. Nutr..

[32] NINDS NET-PD Investigators (2006). A Randomized, Double-Blind, Futility Clinical Trial of Creatine and Minocycline in Early Parkinson Disease. Neurology.

[33] Rawson E.S., Lieberman H.R., Walsh T.M., Zuber S.M., Harhart J.M., Matthews T.C. (2008). Creatine Supplementation Does Not Improve Cognitive Function in Young Adults. Physiol. Behav..

[34] Eckerson J.M., Stout J.R., Moore G.A., Stone N.J., Iwan K.A., Gebauer A.N., Ginsberg R. (2005). Effect of Creatine Phosphate Supplementation on Anaerobic Working Capacity and Body Weight after Two and Six Days of Loading in Men and Women. J. Strength Cond. Res..

[35] Eckerson J.M., Stout J.R., Moore G.A., Stone N.J., Nishimura K., Tamura K. (2004). Effect of Two and Five Days of Creatine Loading on Anaerobic Working Capacity in Women. J. Strength Cond. Res..

[36] Fukuda D.H., Smith A.E., Kendall K.L., Dwyer T.R., Kerksick C.M., Beck T.W., Cramer J.T., Stout J.R. (2010). The Effects of Creatine Loading and Gender on Anaerobic Running Capacity. J. Strength Cond. Res..

[37] Smith A.E., Fukuda D.H., Ryan E.D., Kendall K.L., Cramer J.T., Stout J. (2011). Ergolytic/Ergogenic Effects of Creatine on Aerobic Power. Int. J. Sports Med..

[38] Smith-Ryan A.E., Ryan E.D., Fukuda D.H., Costa P.B., Cramer J.T., Stout J.R. (2014). The Effect of Creatine Loading on Neuromuscular Fatigue in Women. Med. Sci. Sports Exerc..

[39] Stout J.R., Sue Graves B., Cramer J.T., Goldstein E.R., Costa P.B., Smith A.E., Walter A.A. (2007). Effects of Creatine Supplementation on the Onset of Neuromuscular Fatigue Threshold and Muscle Strength in Elderly Men and Women (64–86 Years). J. Nutr. Health Aging.

[40] Walter A.A., Smith A.E., Herda T.J., Ryan E.D., Moon J.R., Cramer J.T., Stout J.R. (2008). Effects of Creatine Loading on Electromyographic Fatigue Threshold in Cycle Ergometry in College-Age Men. Int. J. Sport Nutr. Exerc. Metab..

[41] Van Schuylenbergh R., Van Leemputte M., Hespel P. (2003). Effects of Oral Creatine-Pyruvate Supplementation in Cycling Performance. Int. J. Sports Med..

[42] Camic C.L., Hendrix C.R., Housh T.J., Zuniga J.M., Mielke M., Johnson G.O., Schmidt R.J., Housh D.J. (2010). The Effects of Polyethylene Glycosylated Creatine Supplementation on Muscular Strength and Power. J. Strength Cond. Res..

[43] Camic C.L., Housh T.J., Zuniga J.M., Traylor D.A., Bergstrom H.C., Schmidt R.J., Johnson G.O., Housh D.J. (2014). The Effects of Polyethylene Glycosylated Creatine Supplementation on Anaerobic Performance Measures and Body Composition. J. Strength Cond. Res..

[44] Gao Y., Joshi M., Zhao Z., Mitragotri S. (2024). PEGylated Therapeutics in the Clinic. Bioeng. Transl. Med..

[45] Fielding R.A., Rivas D., Grosicki G.J., Ezzyat Y., Ceglia L., Price L.L., Orhan C., Sahin K., Fowler K., White T. (2021). Effects of Low Doses of L-Carnitine Tartrate and Lipid Multi-Particulate Formulated Creatine Monohydrate on Muscle Protein Synthesis in Myoblasts and Bioavailability in Humans and Rodents. Nutrients.

[46] Liao Q.-Q., Dong Q.-Q., Zhang H., Shu H.-P., Tu Y.-C., Yao L.-J. (2022). Contributions of SGK3 to Transporter-Related Diseases. Front. Cell Dev. Biol..

[47] Ostojic S.M. (2021). Modulation of CT1 Function: From Klotho Protein to Ammonia and Beyond. Front. Nutr..

[48] Escalante G., Gonzalez A.M., St Mart D., Torres M., Echols J., Islas M., Schoenfeld B.J. (2022). Analysis of the Efficacy, Safety, and Cost of Alternative Forms of Creatine Available for Purchase on Amazon.Com: Are Label Claims Supported by Science?. Heliyon.

[49] Kreider R.B., Gonzalez D.E., Hines K., Gil A., Bonilla D.A. (2025). Safety of Creatine Supplementation: Analysis of the Prevalence of Reported Side Effects in Clinical Trials and Adverse Event Reports. J. Int. Soc. Sports Nutr..

[50] Cooper R., Naclerio F., Allgrove J., Jimenez A. (2012). Creatine Supplementation with Specific View to Exercise/Sports Performance: An Update. J. Int. Soc. Sports Nutr..

