# Supplementary material for: Methodological Approaches to Bioavailability of Oral Creatine in Randomized Controlled Trials: A Meta-Research Study
Source: Nutrients. 2026 Aug 18;18(16):2697. doi: 10.3390/nu18162697 (PMC13516631; doi:10.3390/nu18162697)
Supplement: Supplementary file 1 [file nutrients-18-02697-s001.zip › nutrients-4452335-S1.pdf]

Methodological approaches to bioavailability of oral creatine in randomized controlled trials: a meta-research study protocol

Viljemka Bučević Popović, Antonela Sovulj, Sanda Raić, Ivana Pezelj, Malgorzata Bala, Livia Puljak

Introduction

Creatine is a naturally occurring amino acid derivative with an important role in energy metabolism. In the human body, creatine is mostly present in the form of phosphocreatine which serves to rapidly regenerate ATP from ADP and phosphate (Pi). Consequently, the main creatine stores are found in tissues that have a high and fluctuating need for ATP such as skeletal or cardiac muscle and brain [1].

Approximately half of the daily needs for creatine is acquired by synthesis from amino acids arginine and glycine, while the remainder is obtained through the diet mainly from meat and fish [2]. In the past decades, creatine nutritional supplements emerged as alternative sources of creatine to replenish body stores and increase muscle performance in sports [3]. In recent years, studies have reported additional health benefits of creatine in various human diseases and age-related health conditions [1]. For example, multiple studies have explored the benefits of creatine to improve cognition [4], women's health [5], glucose management and diabetes [6], vascular health [7] and immune responses [8].

The most widely used creatine supplement is creatine monohydrate (CrM) [2]. However, evidence suggests that oral bioavailability of CrM is inadequate so that only a fraction of relatively high daily doses administered is actually absorbed in gastrointestinal tract [9]. Thus, novel formulations were developed with the intent to obtain products of improved efficacy and safety [2].

We have shown previously that the issue of bioavailability of bioactive compounds is not necessarily well addressed in the highest level of evidence in medicine. Our studies of curcumin, which is characterized by low oral bioavailability, have shown that researchers rarely address and incorporate in the methodology the issue of bioavailability when conducting clinical trials and systematic reviews [10,11]. We were unable to find such methodological studies for creatine supplements in the available literature, therefore we plan this study to address this research gap.

The aim of this meta-research study is to analyze whether randomized controlled trials (RCTs) on creatine address bioavailability of creatine supplements, whether trialists attempted to use methods that could improve bioavailability, and whether they discussed their results in terms of creatine bioavailability.

Methods

Study design

This is a meta-research study in which we will include published RCTs. Since this study will analyze publicly available research articles, approval of a research protocol by an institutional ethics committee is not applicable.

Inclusion criteria

We will include RCTs published as full reports with results in scholarly journals where creatine was used via oral ingestion for systemic absorption, analyzed as an intervention or a comparator, regardless of the type of participants and type of outcomes that were used in a trial. We will also include RCT protocols, if they were published as a full-text article in a scholarly journal. We will include manuscripts that report *post hoc* analyses of previously published RCTs. The unit of analysis will be a study, and not a publication. Thus, if we find multiple reports for a single study, whereas reports could be a published protocol and multiple publications, we will collate them with the main publication.

Exclusion criteria

We will exclude studies where mixtures of creatine with other compounds were used as an intervention unless other bioactive compounds were administered to both the intervention and control groups, or additional compounds were added for bioavailability enhancement.

We will exclude studies where creatine was used via modes of administration other than oral ingestion for systemic absorption. We will exclude in silico, in vitro and in vivo studies. We will exclude studies published as conference abstracts, results published in editorials and short reports (i.e. not full-text articles in scholarly journals). We will exclude studies available only as record on trial registry without full journal publication of the protocol.

Search and screening

We will search MEDLINE and Embase via Ovid by using the following search syntax: creatine OR creatinol OR creatyl. All bibliographic records found with this search will be retrieved. We will use Covidence online platform (www.covidence.org) for screening. We will upload search results from all sources and remove duplicates using the Covidence algorithm. Furthermore, we will use Cochrane Randomized Controlled Trial (RCT) classifier built into the Covidence, to filter out the studies that are not RCTs. The classifier assesses all the studies imported into Covidence and automatically assigns one of two tags: “Possible RCT” or “Not RCT”. We will

exclude records classified as “not RCT” and then we will screen all the records classified with the ‘Possible RCT’ tag to assess whether they meet the inclusion criteria.

In the first stage of screening, two authors will independently screen titles and abstracts of all records to verify that they indeed fulfill inclusion criteria. In the second stage of screening, we will retrieve full texts of all RCTs that were deemed eligible or potentially eligible in the first screening stage and assess their eligibility against inclusion criteria. Two authors will independently screen each full text. Reasons for excluding records from the study will be noted. In case of disagreement during screening of full texts, two authors will resolve discrepancies in opinion via discussion, or if necessary third author will be included in decision-making.

Data extraction

Two authors will participate in data extraction for each study by independently extracting the data. For each RCT, we will extract the following information:

- the last name of the first author
- year of publication
- the title of study
- trial registration number
- number of participants randomized
- number of study arms
- duration of follow-up
- type of participants included
- type of creatine product that was tested (either as an intervention or a comparator), and a complete description of creatine from the methods.

We will also extract any mentions about the bioavailability of creatine throughout the manuscript, together with the manuscript section where considerations regarding creatine bioavailability were mentioned.

For the type of creatine that was used, we will extract information for both commercial and generic names of the product, as described in the article.

For matching pairs of published RCTs and their corresponding protocols, if we find such cases, we will compare whether there were any differences in the description of creatine, or descriptions of its bioavailability.

After data extraction, we will categorize responses, and present data narratively.

Statistics

Data will be presented as numbers and frequencies, median and interquartile range. Microsoft Excel (Microsoft Inc., Redmond, WA, USA) will be used for analyses.

References

1. Kreider, R.B.; Stout, J.R. Creatine in Health and Disease. *Nutrients* **2021**, *13*, 447, doi:10.3390/nu13020447. [6]
2. Kreider, R.B.; Jäger, R.; Purpura, M. Bioavailability, Efficacy, Safety, and Regulatory Status of Creatine and Related Compounds: A Critical Review. *Nutrients* **2022**, *14*, 1035, doi:10.3390/nu14051035. [2]
3. Cooper, R.; Naclerio, F.; Allgrove, J.; Jimenez, A. Creatine Supplementation with Specific View to Exercise/Sports Performance: An Update. *J. Int. Soc. Sports Nutr.* **2012**, *9*, 33, doi:10.1186/1550-2783-9-33. [50]
4. Smith, A.N.; Morris, J.K.; Carbuhn, A.F.; Herda, T.J.; Keller, J.E.; Sullivan, D.K.; Taylor, M.K. Creatine as a Therapeutic Target in Alzheimer's Disease. *Curr. Dev. Nutr.* **2023**, *7*, 102011, doi:10.1016/j.cdnut.2023.102011. [8]
5. Smith-Ryan, A.E.; Cabre, H.E.; Eckerson, J.M.; Candow, D.G. Creatine Supplementation in Women's Health: A Lifespan Perspective. *Nutrients* **2021**, *13*, 877, doi:10.3390/nu13030877. [9]
6. Solis, M.Y.; Artioli, G.G.; Gualano, B. Potential of Creatine in Glucose Management and Diabetes. *Nutrients* **2021**, *13*, 570, doi:10.3390/nu13020570. [10]
7. Clarke, H.; Hickner, R.C.; Ormsbee, M.J. The Potential Role of Creatine in Vascular Health. *Nutrients* **2021**, *13*, 857, doi:10.3390/nu13030857. [11]
8. Bredahl, E.C.; Eckerson, J.M.; Tracy, S.M.; McDonald, T.L.; Drescher, K.M. The Role of Creatine in the Development and Activation of Immune Responses. *Nutrients* **2021**, *13*, 751, doi:10.3390/nu13030751. [12]
9. Miller, D.W.; Augustine, S.; Robinson, D.H.; Vennerstrom, J.L.; Wagner, J.C. Oral Bioavailability of Creatine Supplements. In *Nutrition and Enhanced Sports Performance*; Elsevier, 2013; pp. 395–403 ISBN 978-0-12-396454-0. [17]
10. Mimica, B.; Bučević Popović, V.; Banjari, I.; Jeličić Kadić, A.; Puljak, L. Methods Used for Enhancing the Bioavailability of Oral Curcumin in Randomized Controlled Trials: A Meta-Research Study. *Pharmaceuticals* **2022**, *15*, 939, doi:10.3390/ph15080939. [21]
11. Bučević Popović, V.; Karahmet Farhat, E.; Banjari, I.; Jeličić Kadić, A.; Puljak, L. Bioavailability of Oral Curcumin in Systematic Reviews: A Methodological Study. *Pharmaceuticals* **2024**, *17*, 164, doi:10.3390/ph17020164. [22]

NOTE: The number in red denotes the reference number in the main manuscript file.
