# Supplementary material for: Methodological Approaches to Bioavailability of Oral Creatine in Randomized Controlled Trials: A Meta-Research Study
Source: Nutrients. 2026 Aug 18;18(16):2697. doi: 10.3390/nu18162697 (PMC13516631; doi:10.3390/nu18162697)
Supplement: Supplementary file 1 [file nutrients-18-02697-s001.zip › Supplementary Table S4.pdf]

Supplementary Table S4. Studies that compared multiple creatine products (creatine monohydrate vs. alternative creatine form).

Study	Products Compared (dose)	Study Aim	Conclusions Regarding Bioavailability (Verbatim Quotes)
Askow 2022 [25]	creatine monohydrate (5 g/d)<br>creatyl-L-leucine (5 g/d)	To compare the effects of creatine supplementation creatine on muscle creatine content	Creatine monohydrate supplementation significantly increased muscle Cr content by ~24%, whereas there was no statistically significant change in muscle Cr content in the placebo and/or the creatyl-L-leucine conditions.
Galvan 2016 [26]	creatine monohydrate<br>*Study 1:* 5 g/d *Study 2:* 12 (loading) or 3 g/d (maintenance)<br>creatine nitrate<br>*Study 1:* LOW 1.5 g/day or HIGH 3 g/d<br>*Study 2:* LOW 6 (loading) or 1.5 (maintenance) g/d<br>HIGH 12 g (loading) or 3 (maintenance) g/d	To examine the dose-dependent effects of creatine nitrate on acute and chronic indices of safety and exercise performance compare it with creatine monohydrate	There was no improvement in cycling anaerobic power output for our assessments despite the creatine monohydrate group having significantly greater creatine stores than creatine nitrate-Low, creatine nitrate-High, and placebo groups.<br>These findings contrast claims that there is no need to load when taking creatine nitrate due to greater solubility and retention.<br>There was no evidence that creatine nitrate at recommended or twice recommended doses is more efficacious than creatine monohydrate at the doses studied.
Herda 2009 [27]	creatine monohydrate (5 g/d)<br>polyethylene glycosylated (PEG) creatine hydrochloride (doses equivalent to 1.25 g/d or 2.50 g of creatine monohydrate)	To examine the effects of a moderate dose of creatine monohydrate and two smaller doses of PEG creatine on muscular strength, endurance, and power output	Creatine monohydrate supplementation resulted in increases in body mass and absolute mean power that were significantly greater than those for the placebo, PEG1.25 and PEG2.50 groups.<br>PEG creatine may result in similar increases in strength when compared with CM but with a dose reduction of as much as 75%.
