# Supplementary material for: Methodological Approaches to Bioavailability of Oral Creatine in Randomized Controlled Trials: A Meta-Research Study
Source: Nutrients. 2026 Aug 18;18(16):2697. doi: 10.3390/nu18162697 (PMC13516631; doi:10.3390/nu18162697)
Supplement: Supplementary file 1 [file nutrients-18-02697-s001.zip › Supplementary Table S5.pdf]

Supplementary Table S5. Molar quantities of creatine used in creatine trials comparing various creatine forms. The following values were used for calculations: Mw (creatine monohydrate) = 149.15 g/mol, Mw (creatyl-L-leucine) = 244 g/mol and Mw (creatine nitrate) = 194.15 g/mol

Study	Creatine forms	Daily dose of creatine supplement	Molar quantities of creatine contained	Moles of creatine delivered with creatine monohydrate/moles of creatine delivered with alternative formulation
Askow 2022 [25]	creatine monohydrate<br>creatyl-L-leucine	5 g<br>5 g	33.52 mmol<br>20.49 mmol	**1.6**
Galvan 2016 [26]	*Study 1:*<br>creatine monohydrate<br>creatine nitrate	 5 g<br>1.5 or 3 g	 33.52 mmol<br>7.73 or 15.45 mmol	**4.3 or 2.2**
	Study 2:<br>Loading phase<br>creatine monohydrate<br>creatine nitrate	 <br>12 g<br>6 or 12 g	 <br>80.46 mmol<br>30.90 or 61.81 mmol	**2.6 or 1.3**
	Maintenance phase<br>creatine monohydrate<br>creatine nitrate	 3 g<br>1.5 or 3 g	 20.11 mmol<br>7.73 or 15.45 mmol	**2.6 or 1.3**
Herda 2009 [27]	creatine monohydrate<br>polyethylene glycosylated (PEG) creatine hydrochloride	5 g<br>equivalent to 1.25 g/d or 2.50 g of creatine monohydrate	33.52 mmol<br>8.38 or 16.76 mmol	**4 or 2**
